# Methylation of the Glucocorticoid Receptor Gene in Children with Somatic Symptom Disorder: A Case-Control Study

**DOI:** 10.3390/epigenomes9020022

**Published:** 2025-06-13

**Authors:** Kyoko Hatta, Masato Kantake, Kyoko Tanaka, Hirofumi Nakaoka, Toshiaki Shimizu, Hiromichi Shoji

**Affiliations:** 1 Department of Pediatrics, Juntendo University Faculty of Medicine, Tokyo 113-0033, Japan; 2 Department of Neonatology, Juntendo University Nerima Hospital, 3-1-10 Nerima Takanodai, Nerima-ku, Tokyo 177-8521, Japan; 3 Department of Psychosocial Medicine, National Center for Child Health and Development, Tokyo 157-8535, Japan; 4 Department of Cancer Genome Research, Sasaki Institute, Sasaki Foundation, Tokyo 101-0062, Japan

**Keywords:** glucocorticoid receptor, NR3C1, methylation, hypothalamic–pituitary–adrenal axis, somatic symptom disorder

## Abstract

**Background:** Somatic symptom disorder (SSD) in children may be influenced by stress reactivity and psychosocial factors. The glucocorticoid receptor (GR), encoded by NR3C1, is a key mediator of stress responses. However, the relationship between NR3C1 methylation and SSD remains unclear. **Methods:** We analyzed NR3C1 exon 1F methylation in cell-free DNA from saliva in 34 children with SSD and 29 age- and sex-matched controls using bisulfite amplicon sequencing. Psychological assessments included the Beck Depression Inventory-II (BDI-II) and KINDL questionnaires to evaluate associations with methylation patterns. **Results:** Methylation levels showed age-related differences. In children under 13, CpG sites displayed mixed methylation, and specific sites correlated with KINDL and BDI-II scores. KINDL physical and total well-being scores negatively correlated with CpG30 and positively with CpG35; BDI-II scores negatively correlated with CpG32 and CpG35. In children aged 13 or older, CpG sites showed uniformly high methylation with no correlation to psychological measures. The SSD group showed significantly higher average methylation across the exon 1F region than controls in the older age group. These children also had more cases of orthostatic dysregulation and longer illness duration. **Conclusions:** This study suggests age-dependent epigenetic regulation of NR3C1 in SSD. While younger children showed CpG-specific correlations with psychological symptoms, older children demonstrated uniformly high methylation and potentially reduced gene expression, potentially reflecting cumulative stress, autonomic dysfunction, and internalizing disorders such as anxiety and depression.

## 1. Introduction

Somatic symptom disorder (SSD) has been defined as a stress-related disorder [1]. Early life stress, such as childhood maltreatment, early parental death, and childhood trauma, is a well-known psychosocial risk factor for SSD [2,3]. Moreover, short-lasting stressful periods are identified as the most frequently reported triggers of SSD [4]. Thus, stress vulnerability plays an important role in the pathogenic mechanism of this disorder. The glucocorticoid receptor (GR) gene, the nuclear receptor subfamily 3 group C member 1 (NR3C1), is associated with stress vulnerability via the hypothalamic–pituitary–adrenal (HPA) axis, which is considered the primary stress response system of the body and is regulated by the hypothalamic GR through a negative feedback loop [5]. Weaver et al. identified an epigenetic modification of the NR3C1 gene indicative of differential maternal rearing behavior in rodents [6]. Low levels of maternal care result in greater methylation of the GR, leading to long-lasting decreases in GR expression [7].

In humans, prenatal stressors such as maternal depression and anxiety are associated with NR3C1 methylation, particularly in the exon 1F region, the promoter region for the gene [8,9,10]. Early life stress (e.g., child abuse and trauma) and conditions such as depression, bipolar disorder, and post-traumatic stress disorder are also associated with NR3C1 methylation [7,11,12,13,14]. Furthermore, NR3C1 methylation is positively associated with internalizing behavior problems in children [7]. Vertebrate genomes are often methylated at CpG dinucleotides. CpG-poor genomic regions are interrupted by CpG islands [1], which are predominantly nonmethylated and typically occur near transcription start sites [15]. A total of 39 CpG sites have been identified in the NR3C1 exon 1F CpG island. Among these, CpG16–18, 30–32, and 37–38 are essential regions that act as binding sites for nerve growth factor-inducible protein A (NGFI-A), a central regulator of early inflammatory and immune processes. Furthermore, NGFI-A potentiates NR3C1 1F promoter activity.

Because NR3C1 methylation is associated with SSD risk factors such as childhood maltreatment and stress vulnerability, we hypothesized that patients with SSD may present with higher rates of NR3C1 methylation. However, there are no reports on the association between NR3C1-1F methylation and SSD in adults or children. This study thus aimed to determine the association between NR3C1-1F methylation and SSD in children.

## 2. Results

### 2.1. Participant Characteristics

This study included 34 children in the SSD group and 29 in the control group. Characteristics of the participants are outlined in Table 1 and Table 2. Three control children were excluded after being diagnosed with SSD, but they were not added to the SSD group due to the absence of a formal diagnostic interview. According to the Diagnostic and Statistical Manual of Mental Disorders, Fifth Edition (DSM-5) severity criteria, 13 SSD patients had severe symptoms, 7 had moderate symptoms, and 14 had mild symptoms [16]. Among the SSD diagnoses, 26 involved irritable bowel syndrome, 25 orthostatic dysregulation (OD), 4 chronic headache, and 1 fibromyalgia syndrome. Stratification by age revealed that younger SSD participants (<13 years) had significantly more OD cases with illness duration exceeding 3 years (*p* = 0.016 and *p* = 0.023, respectively). Of the SSD group, 18 (52.9%) were female, with a mean age of 13.4 ± 1.9 years, compared to 11 females (37.9%) in the control group (mean age 12.7 ± 1.9 years). No significant sex or age differences were found (*p* = 0.22 and *p* = 0.23, respectively). Two SSD participants had depressive disorders, two had anxiety disorder, two had ASD, and two had ADHD. One participant used risperidone (age 11), and five used aripiprazole (ages 13, 10, 10, 12, and 12). No control participants had psychiatric diagnoses or took psychiatric medications. The SSD group showed a higher incidence of familial mental health issues (*p* < 0.01), including SSD (n = 3), major depressive disorder (n = 2), bipolar disorder (n = 3), adjustment disorder (n = 2), and ASD (n = 1). In contrast, only two cases of adjustment disorder were reported in the control group.

### 2.2. GR Gene Methylation Rate

**Figure 1:**
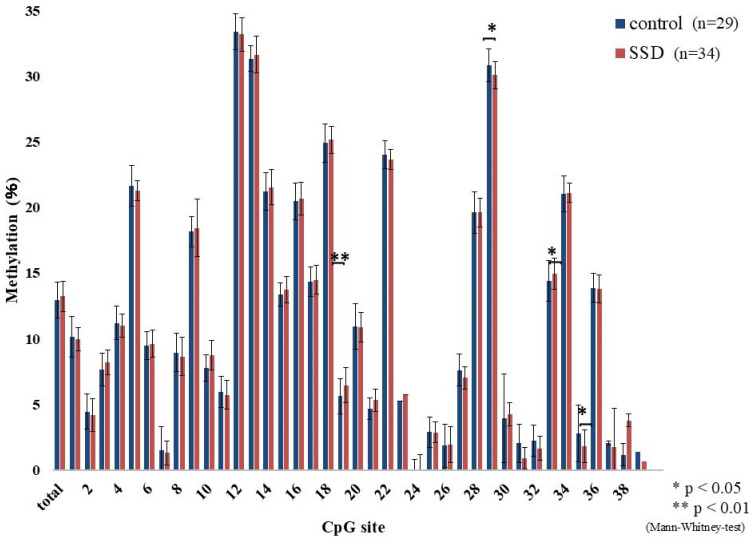
Percent methylation at each CpG site.

**Figure 2:**
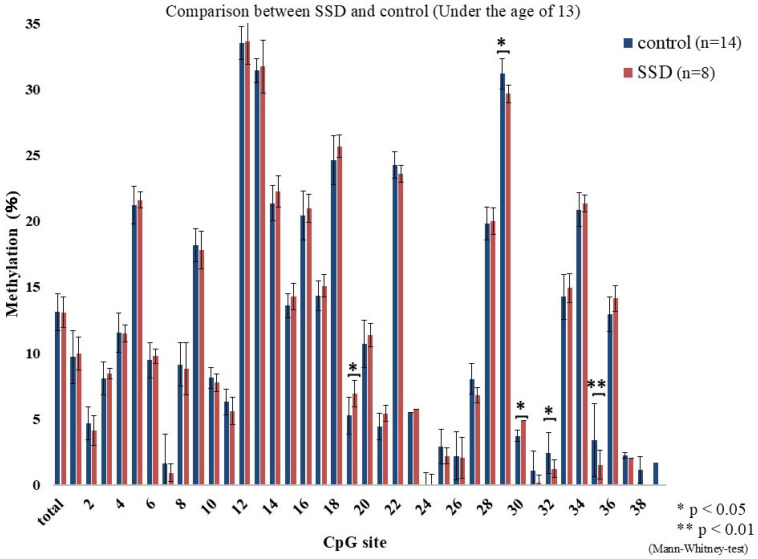
Percent methylation at each CpG site (under the age of 13).

**Figure 3:**
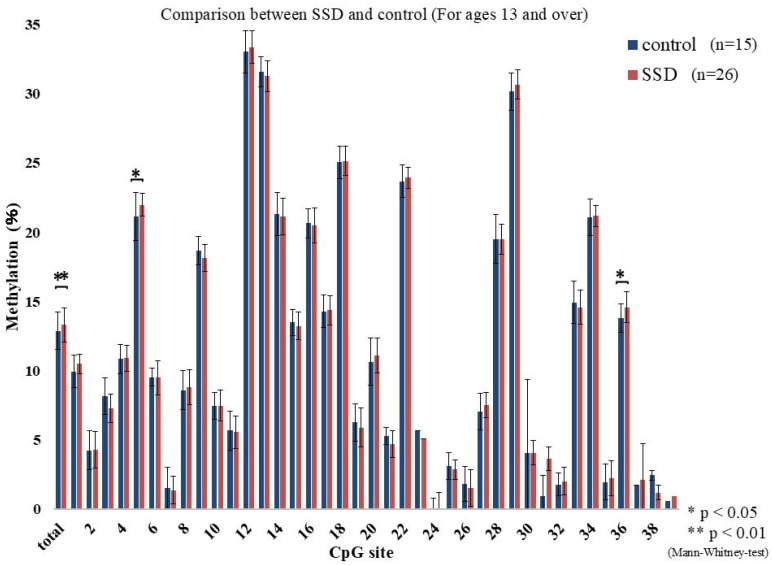
Percent methylation at each CpG site (for ages 13 and over).

**Figure 4:**
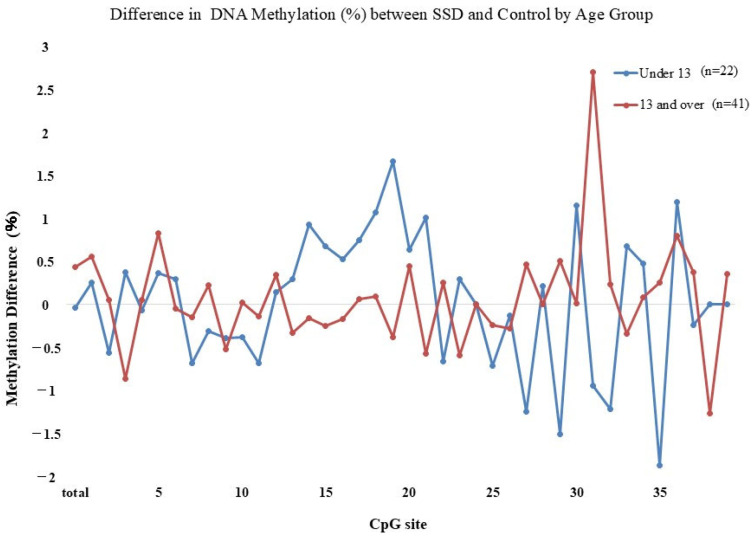
Difference in DNA methylation (%) between SSD and control by age group.

The methylation percentages across CpG sites are shown in Figure 1. Differences between the SSD and control groups were found at CpG 19 (*p* = 0.009), CpG 29 (*p* = 0.023), CpG 33 (*p* = 0.038), and CpG 35 (*p* = 0.035). These showed a mosaic pattern with both hyper- and hypomethylated sites. Considering the school system transition at age 13 in Japan, participants were divided into under 13 and 13 or older. Among those under 13, CpG 19 (*p* = 0.016), CpG 29 (*p* = 0.02), CpG 30 (*p* = 0.016), CpG 32 (*p* = 0.028), and CpG 35 (*p* = 0.001) differed significantly between groups, again with mosaic characteristics (Figure 2). In those 13 or older, CpG 5 (*p* = 0.017) and CpG 36 (*p* = 0.038) showed elevated methylation in SSD participants and lacked the mosaic variability (Figure 3). The average methylation rate was also significantly higher in SSD participants aged 13 and older (*p* = 0.001). Figure 4 displays these patterns across age groups.

### 2.3. BDI-II and KINDL Scores

Group comparisons of BDI-II and KINDL scores are presented in Table 3. SSD participants had significantly higher BDI-II scores (mean 16.7) compared to controls (mean 6.7; *p* = 0.001). Similarly, KINDL total, physical well-being, emotional well-being, friend, and school subscales showed significantly lower scores in the SSD group (*p* = 0.001, *p* = 0.001, *p* = 0.032, *p* = 0.016, and *p* = 0.048, respectively).

### 2.4. Correlations Between GR Methylation Rate and Symptomatology

In children under 13, CpG 19 and 30 showed negative correlations with KINDL total and physical well-being scores (r = −0.46 and −0.53). CpG 29 had a positive correlation with KINDL physical well-being (r = 0.49). CpG 32 and 35 positively correlated with total KINDL scores (r = 0.52 for both) and negatively with BDI-II scores (r = −0.47 and −0.63). CpG 32 also positively correlated with the KINDL friends score (r = 0.60) (Table 4). No significant correlations were found in the 13-and-older group (Table 5).

## 3. Materials and Methods

### 3.1. Participants

We conducted a case-control study including 63 children aged 9–16 years. The SSD group consisted of 34 individuals meeting DSM-5 criteria for SSD, recruited from Juntendo University Hospital and the National Center for Child Health and Development between 2019 and 2020. According to the Japanese Society of Psychosomatic Pediatrics, physical conditions classified as SSD include irritable bowel syndrome, orthostatic dysregulation (OD), chronic headache, and fibromyalgia. Diagnoses were made by pediatricians specializing in child psychiatry. Exclusion criteria were a history of serious head injury or psychosis. A control group of 32 age-matched children with typical development was recruited from local elementary and junior high schools during the same time period. Information sessions were held for parents and children, and written informed consent was obtained. Children reporting somatic symptoms or with diagnosed psychiatric or neurodevelopmental disorders were excluded from the control group.

### 3.2. Procedure

All participants completed the Beck Depression Inventory-II (BDI-II) and KINDL questionnaires, and SSD severity was evaluated using DSM-5 criteria. Participants completed the assessments independently.

The BDI-II is a 21-item self-report questionnaire measuring cognitive, somatic, and emotional symptoms of depression over the past two weeks [17], with responses scored on a 4-point scale (0–3). Higher scores indicate more severe depressive symptoms [18]. The Japanese version has shown good test–retest reliability and internal consistency (Cronbach’s α = 0.87) [19].

The KINDL questionnaire assesses health-related quality of life (QOL) and consists of 24 items across six subscales: physical well-being, emotional well-being, self-esteem, family, friends, and school. Responses use a 5-point scale, with higher scores reflecting better QOL [20,21]. We administered the Kid-KINDL (for ages 8–12) and Kiddo-KINDL (for ages 13–16) Japanese versions, which demonstrate solid reliability and validity [22,23].

Demographic data such as age, sex, medication, and history of bullying or abuse were collected. Parents provided information on perinatal complications, hospitalizations, socioeconomic status, family mental health history, and parental separation. Psychiatric diagnoses in family members were limited to those confirmed at medical institutions. The study was approved by the Ethics Committee of Juntendo University (Approval No. 2019013).

### 3.3. DNA Methylation Analysis

Prior studies report moderate correlation between NR3C1 methylation levels in saliva and blood, suggesting saliva is a noninvasive alternative for pediatric studies [24,25]. Saliva was collected using Oragene-DNA kits (DNA Genotek Inc., Ottawa, ON, Canada) and stored at room temperature. DNA was extracted using the DNA Extractor SP Kit (Wako Pure Chemical Industries, Tokyo, Japan). Bisulfite conversion was performed using the EZ DNA Methylation-Direct Kit (Zymo Research, Irvine, CA, USA). A 5 μL aliquot of each bisulfite-treated sample was amplified targeting the NR3C1 exon 1F promoter region, following protocols in the supplemental file. PCR products were purified via gel extraction and stained with ViewaBlue Stain KANTO (Kanto Chemical Co., Tokyo, Japan).

Methylation rates were calculated using the Mquant method (Leakey et al.) [26]. Using ImageJ 1.53 software (NIH, Bethesda, MD, USA), the average peak height of ten T residues (five on each side of the CpG site) was calculated. Each peak had to exceed the background signal by at least 10-fold. The delta T (difference between peak T and mean T) was used to derive methylation percentage as (delta T)/(T average).

A total of 39 CpG sites within exon 1F (−3466 to −3189 bp upstream of the ATG start site) were analyzed, including cg14278308, cg142783622, cg142783628, cg142783638, cg142783640, cg142783656, cg142783664, cg142783679, cg142783686, cg142783689, cg142783703, cg142783713, cg142783717, cg142783731, cg142783736, cg142783743, cg142783745, cg142783756, cg142783767, cg142783769, cg142783772, cg142783775, cg142783778, cg142783781, cg142783786, cg142783793, cg142783810, cg142783822, cg142783832, cg142783838, cg142783844, cg142783849, cg142783854, cg142783858, cg142783860, cg142783864, cg142783870, cg142783874, and cg142783884.

### 3.4. Statistical Analysis

All results are reported as the mean ± standard deviation (SD). Fisher’s exact test and Mann–Whitney U-tests were applied to compare categorical and continuous variables between groups, as data were not normally distributed in the Kolmogorov–Smirnov test, quantile–quantile plot, and histogram. Correlations were assessed using Spearman’s rho. Logistic regression controlling for sex and age was also conducted [27,28]. Statistical analyses were performed using SPSS version 7.0 for Windows, and significance was set at *p* < 0.05.

## 4. Discussion

To our knowledge, this is the first investigation to examine NR3C1 methylation in children with SSD through both biological and psychological assessments. We adopted a noninvasive, rapid, and efficient method, suitable for future population-based research.

Although differences in methylation were detected between groups, these alterations presented as a mosaic pattern, with a mixture of hypermethylated and hypomethylated CpG positions. As a result, we divided the analysis based on participant age: those under 13 and those 13 or older.

Among children younger than 13 years, significant methylation differences at CpG 19 (SSD > control), CpG 29 (SSD < control), CpG 30 (SSD > control), CpG 32 (SSD < control), and CpG 35 (SSD < control) were found. There was no group difference in the overall methylation average (*p* = 0.87). In participants aged 13 and above, group differences appeared at CpG 5 and CpG 36 (both SSD > control), and methylation levels were generally high and uniform. Total methylation was significantly higher in this older SSD group (*p* = 0.001).

Watkeys’ systematic review indicates that DNA methylation within exon 1F is inversely associated with NR3C1 expression. Specific CpG sites (e.g., 4, 6, 7, 18, 23, 29, 34, 35, and 37) are negatively correlated with gene transcription [29]. Based on the current data, lower methylation at CpG 29 and 35 in younger SSD participants may imply higher GR expression. In contrast, adolescents showed increased overall methylation without specific site differences, which might reflect reduced NR3C1 expression. These findings point to possible age-related bidirectional epigenetic control around age 13.

Additionally, prior studies have reported links between NR3C1 methylation and mental disorders. Higher average methylation across exon 1F has been associated with internalizing symptoms. CpGs 1, 29–32, and 35–38 have all been implicated, with CpG 38 particularly elevated in depression. Anxiety has been associated with greater methylation at CpGs 12, 21, 30, 31, and 32 [29].

In our study, younger children displayed heterogeneous methylation, suggesting both increases and decreases depending on the site. These variations may affect gene expression but are not clearly tied to SSD in this age group. CpG 36, which differed significantly in older children, is noteworthy—it has been associated with psychiatric symptoms and responds to both cortisol and military stress exposure [14,30]. CpG 5 is typically hypomethylated in PTSD, which is known to have reduced HPA axis activity. In the present study, it showed higher methylation in SSD adolescents, suggesting that HPA axis activity may differ in SSD from PTSD.

Lower NR3C1 expression impairs HPA axis negative feedback, prolonging cortisol release and increasing stress sensitivity. In SSD, this may underlie persistent physical complaints like fatigue or pain and also contribute to emotional dysregulation and vulnerability to comorbid mental health problems.

CpG 30, which was significantly altered in younger SSD participants, is a canonical NGFI-A binding site. This site’s methylation level was linked with KINDL scores, and SSD is well known to reduce quality of life. These findings suggest that CpG-specific methylation may influence symptom presentation [31,32,33].

Whereas adult SSD is often associated with hypomethylation [34], our pediatric results showed the opposite trend. Based on these results, we considered the following hypotheses. First, a prolonged disease period may be required for hypomethylation to be observed in SSD. In the current study, changes in hypermethylation were observed in patients who had the disorder for >3 years, whereas a mosaic condition was observed in patients who had been diagnosed for <3 years. These findings suggest that methylation in children with SSD may change over time. However, in adults, the disease may last longer; therefore, methylation may gradually change from a hyper to a hypo state. In patients with adverse childhood experiences (ACEs), cardiac reactivity increases during the acute phase of stress and decreases over time [35]. Even in children with SSD, the symptoms of SSD may become a stress factor and cause similar changes.

It is also plausible that hypermethylation occurs in SSD patients without ACEs. Many previous studies focus on SSD with ACE exposure [36], but no clear group differences in ACEs were found in our data. Thus, stressors other than ACEs may drive methylation.

Another hypothesis concerns orthostatic dysregulation (OD), which was more frequent in SSD participants. Although NR3C1 methylation in OD has not been studied, research linking OD to HPA axis dysfunction and ACEs supports this pathway. Dempster proposed that ACEs might influence OD through reduced GR expression [35]. Although our study was limited in size, related findings have been reported in individuals with functional neurological symptoms, showing elevated HPA axis function [35].

Furthermore, the transition from mosaic to hypermethylated states observed around adolescence may be shaped more by puberty itself than illness duration. Puberty is a well-known critical window for mental health vulnerability and epigenetic reprogramming. Findings from the TRAIL study support this. Even in preadolescents, we observed methylation correlating with psychological test scores, suggesting that psychosocial stress is active prior to puberty. Continued stress exposure through adolescence, combined with increased vulnerability, might promote a shift toward a stable hypermethylated profile. The decreasing effectiveness of maternal care in adolescence may also reduce resilience, reinforcing the need for longitudinal follow-up to monitor these changes and identify potential protective factors.

Previous studies have demonstrated that methylation may be reversible through successful treatment or medication. Therefore, early identification and intervention for SSD symptoms are vital to prevent persistent NR3C1 hypermethylation.

Additionally, our study found higher rates of familial mental illness among SSD participants. These may influence a child’s stress sensitivity through both genetic and environmental pathways [37,38,39,40,41,42]. Further research is needed to clarify the complex interaction between family mental health and NR3C1 methylation in children.

This study has several limitations. First, it included a combination of patients with different types of SSD, and the sample size of the SSD and control groups was small. Due to the small sample size, confounding factors such as the home environment could not be adjusted for. Therefore, it is possible that statistical analyses accounting for these confounders may yield different correlations than those observed in the present study.

Although, as mentioned earlier, a number of disorders are considered as SSD, the pathological condition and the effect on methylation of each disease may vary; thus, additional studies should recruit more participants to evaluate the features and longitudinal changes of each disease and the association between the risk factors for SSD, such as economic status and ACEs. Second, several studies have indicated the hereditary aspect of methylation, and SSD is a well-known hereditary disease. Therefore, studies using a familial approach to clarify the chronological relationship between 1F methylation and SSD are warranted.

Third, the limited magnitude of methylation changes observed, along with the absence of gene expression analysis, represents a key limitation of the present study. While several studies have utilized saliva and, as previously noted, correlations with blood samples have been observed, this nonetheless represents a limitation in cross-study comparisons [24,25]. Moreover, the absence of data on oral health conditions—such as dental caries and periodontal disease—that may be associated with methylation profiles is considered a limitation of this study. Future investigations incorporating a larger sample size and parallel assessments of gene expression will be essential to elucidate how the age-related differences in methylation identified here may contribute to transcriptional regulation.

The findings of this study suggest that children with SSD are more likely to undergo changes in NR3C1 methylation caused by psychological stress. Future studies focused on disease duration and type will help further elucidate the pathology of SSD, identify protective factors, and discover new treatments.

## 5. Conclusions

This study is the first to examine NR3C1 exon 1F methylation in children diagnosed with SSD. It revealed that methylation patterns in this region vary by developmental stage. In younger children, methylation at specific CpG sites correlated with depressive symptoms and quality of life measures, suggesting the influence of psychological stress on epigenetic regulation early in life.

In adolescents, a different pattern emerged—widespread hypermethylation without site-specific psychological associations. This may reflect cumulative stress or neurobiological adaptations during puberty. Furthermore, the elevated rate of OD and prolonged illness in this group supports the idea that chronic stress and physiological dysregulation reinforce these methylation changes. Together, these findings support the role of age-dependent epigenetic modifications in SSD and highlight the importance of early detection and targeted interventions.

## Figures and Tables

**Table 1:** Sample characteristics.

Indicators	SSD (n = 34)	Controls (n = 29)	*p*-Value (Mann–Whitney Test) (Fisher’s Exact Test)
Age, mean (SD) years	13.38 (1.88)	12.71 (1.86)	0.22
Female, n (%)	18 (52.94)	11 (37.93)	0.23
**Household income**			0.38
JPY 0–4,999,999, n (%)	10 (29.41)	11 (37.93)	
JPY 5,000,000–10,000,000, n (%)	11 (32.35)	11 (37.93)	
JPY 10,000,000+, n (%)	9 (26.47)	5 (17.24)	
Perinatal complication, n (%)	2 (5.88)	6 (20.69)	0.09
Hospitalization, n (%)	8 (23.53)	6 (20.70)	0.44
Victim of bullying, n (%)	8 (23.53)	4 (13.79)	0.33
Victim of abuse, n (%)	3 (8.82)	2 (6.90)	0.73
Familial mental health problem, n (%)	13 (38.24)	3 (10.34)	<0.01
Parental divorce, n (%)	5 (14.70)	4 (13.79)	0.69

**Table 2:** Case characteristics.

Indicators	Under Age of 13 (n = 8)	Ages 13 and Over (n = 26)	*p*-Value (Fisher’s Exact Test)
Irritable bowel syndrome, n (%)	7 (88)	18 (69)	0.30
Orthostatic dysregulation, n (%)	3 (38)	22 (85)	0.03
Chronic headache, n (%)	0 (0)	4 (15)	0.32
Fibromyalgia syndrome, n (%)	0 (0)	1 (4)	0.77
Several somatic symptom diseases, n (%)	2 (25)	17 (65)	0.08
Duration of the disease more than three years, n (%)	1 (13)	14 (54)	0.02

**Table 3:** Mean BDI, kid-KINDL and subscale scores (and SDs) based on groups. Mean BDI, kid-KINDL and subscale scores (and SDs): SSD vs controls (Mann–Whitney test).

CpG Site	BDI-II	KINDL Total	Physical Well-Being	Emotional Well-Being	Self-Esteem	Family	Friends	School
**SSD** **(n = 34)**	16.69 (12.16) **	77.16 (12.95) **	11.97 (3.02) *	14.81 (3.28) *	9.94 (3.49)	14.89 (3.56)	14.19 (4.03) *	11.41 (4.15) *
**Controls (n = 29)**	6.70 (5.92) **	91.22 (11.95) **	16.67 (2.73) *	16.81 (3.01) *	11.89 (3.97)	15.59 (3.53)	16.85 (2.78) *	13.41 (2.90) *
***p*** **-Value**	0.001	0.001	0.000	0.03	0.08	0.62	0.02	0.04

Note: * *p* < 0.05; ** *p* < 0.01.

**Table 4:** Correlations of methylation BDI and KINDL: r (*p*-value) (under the age of 13); correlations of methylation, BDI, and KINDL in Children under 13: r (*p*-value).

	BDI-II	KINDL Total	Physical Well-Being	Emotional Well-Being	Self-Esteem	Family	Friends	School
**CpG 19**	0.39 (0.09)	−0.46 (0.04) *	−0.46 (0.04) *	−0.11 (0.66)	−0.09 (0.71)	−0.19 (0.43)	−0.36 (0.12)	−0.30 (0.20)
**CpG 29**	−0.22 (0.35)	0.13 (0.58)	0.49 (0.03) *	−0.25 (0.29)	−0.12 (0.61)	0.20 (0.40)	0.06 (0.81)	−0.14 (0.56)
**CpG 30**	0.37 (0.11)	−0.47 (0.04) *	−0.53 (0.02) *	0.08 (0.75)	−0.33 (0.16)	−0.41 (0.07)	−0.14 (0.56)	−0.33 (0.16)
**CpG 32**	−0.47 (0.04) *	0.52 (0.02) *	0.31 (0.20)	0.28 (0.25)	0.39 (0.10)	0.37 (0.12)	0.60 (0.008) **	0.31 (0.20)
**CpG 35**	−0.63 (0.003) **	0.47 (0.04) *	0.72 (0.00) **	0.26 (0.28)	0.18 (0.46)	−0.03 (0.91)	0.28 (0.23)	0.14 (0.56)

(n = 22) * *p* < 0.05; ** *p* < 0.01. (Spearman’s rho).

**Table 5:** Correlations of methylation BDI and KINDL: r (*p*-value) (for ages 13 and over); correlations of methylation, BDI, and KINDL in Children over 13: r (*p*-value) (n = 41) (Spearman’s rho).

	BDI-II	KINDL Total	Physical Well-Being	Emotional Well-Being	Self-Esteem	Family	Friends	School
CpG 5	0.08 (0.63)	−0.14 (0.40)	−0.21 (0.22)	−0.12 (0.49)	−0.08 (0.62)	−0.11 (0.51)	−0.05 (0.77)	−0.01 (0.93)
CpG 36	−0.02 (0.93)	−0.09 (0.61)	−0.15 (0.39)	−0.26 (0.12)	−0.05 (0.76)	−0.20 (0.23)	0.009 (0.96)	0.05 (0.78)

## Data Availability

The datasets used and/or analyzed during the current study are available from the corresponding author on a reasonable request.

## Abbreviations

SSD: somatic symptom disorder; GR: glucocorticoid receptor; BDI-II: Beck Depression Inventory; ASD: autism spectrum disorder; OD: orthostatic dysregulation
